# Suspected pediatric sleep disordered breathing – when do we perform polysomnography?

**DOI:** 10.1007/s00405-025-09480-z

**Published:** 2025-05-29

**Authors:** Ella Ruikka, Lotta Ukonaho, Ilkka Kivekäs, Maija Katila, Heini Huhtala, Saara Markkanen

**Affiliations:** 1 Department of Otorhinolaryngology, Tampere University Hospital, Wellbeing Services County of Pirkanmaa, Elämänaukio 2, Tampere, 33520 Finland; 2 Faculty of Medicine and Health Technology, Tampere University, Kalevantie 4, Tampere, 33100 Finland; 3 Department of Pediatrics, Tampere University Hospital, Wellbeing Services County of Pirkanmaa, Elämänaukio 2, Tampere, 33520 Finland; 4 Faculty of Social Sciences, Tampere University, Kalevantie 4, Tampere, 33100 Finland

**Keywords:** Sleep disordered breathing, Obstructive sleep apnea, Polysomnography, Adenotonsillar hypertrophy, Pediatric

## Abstract

**Objective:**

Snoring is common in children and diagnosing sleep disordered breathing (SDB) and assessing its severity is unreliable without polysomnography (PSG), a costly procedure that is not always readily available. Our objective was to identify those factors that influence the decision to refer children for PSG.

**Methods:**

A retrospective cohort study of 1267 children aged 0–16 years with suspected SDB and/or adenotonsillar hypertrophy.

**Results:**

A total of 212 children (16.5%) underwent PSG. The likelihood of PSG being performed increased with age (OR 1.14, *p* <.001, 95% CI 1.08–1.20), male sex (OR 1.72, p 0.008, 95% CI 1.15–2.58), and certain OSA symptoms. Children with a moderate (OR 3.82, *p* <.001, 95% CI 2.66–5.57) or severe comorbidity (OR 9.84, *p* <.001, 95% CI 6.48–14.92) were more likely to undergo PSG than healthy individuals. Children referred from pediatric specialties were also more likely to undergo PSG (OR 15.00, *p* <.001, 95% CI 9.76–23.05) than children from primary health care, whereas children referred from a dentist were less likely to do so (OR 0.12, p 0.034, 95% CI 0.02–0.85). Children who underwent PSG were more likely to be treated conservatively than those diagnosed clinically.

**Conclusions:**

The decision to refer children for PSG is susceptible to subjectivity and the factors influencing it are likely broadly generalizable. Therefore, a guideline for referring a child with suspected SDB for PSG is needed. This would not only standardize the SDB diagnostic pathway, but also potentially reduce the need for further follow-up or surgeries.

**Supplementary Information:**

The online version contains supplementary material available at 10.1007/s00405-025-09480-z.

## Introduction

Children with sleep disordered breathing (SDB) make up a large patient group that is commonly treated in the ENT department. The prevalence of habitual snoring in the pediatric population varies among studies, ranging from 3 to 12%. It has been estimated that between 1% and 5.7% of children have obstructive sleep apnea (OSA), with peak prevalence occurring between 2 and 8 years of age [1]. The current understanding is that snoring and OSA are not independent entities but rather a continuum within the SDB spectrum [2, 3]. In this article, we focus solely on the obstructive subtype of the SDB spectrum.

The most commonly recognized risk factor for obstructive childhood SDB is adenotonsillar hypertrophy. However, many other conditions, including childhood obesity, craniofacial abnormalities, inflammatory factors, and neuromuscular diseases, have been identified that contribute to the onset of obstructive SDB [3–6].

SDB cannot be reliably ruled out or diagnosed based solely on caregiver observations. While a clinical history and a physical examination can suggest the likelihood of SDB, they do not provide a definitive diagnosis. Neither can the severity of SDB be reliably assessed based on clinical diagnosis alone [3, 7]. Overnight polysomnography (PSG) is considered the gold standard for diagnosing SDB in children. Based on PSG findings, assessing the need for operative treatment and postoperative monitoring is made easier. When PSG is not available, alternative diagnostic modalities, such as ambulatory PSG, polygraphy, nocturnal oximetry, or pediatric sleep questionnaires, can be considered, though they cannot substitute for PSG [8, 9].

Adenotonsillectomy is the gold standard for the treatment of obstructive SDB and is an effective treatment modality for children with OSA, reducing symptoms and improving quality of life and PSG findings [10–12]. The popularity of partial tonsillectomy as an effective treatment for OSA has increased significantly in recent years [13–16].

Some children with SDB have their PSG findings normalize within a few months of watchful waiting [10, 12]. However, children with moderate-to-severe OSA are less likely to have their PSG findings normalize or to experience symptom resolution without treatment [8, 9, 17, 18]. This highlights the importance of accurately determining the severity of OSA using PSG.

There are previous guidelines regarding when children with suspected SDB should undergo PSG [1, 8, 9, 17, 19, 20]. For example, the American Academy of Pediatrics guideline [1] recommends always obtaining PSG if a child is reported to snore on a regular basis and has other OSA symptoms or OSA findings upon physical examination. At the very least, the child should be referred for further evaluation and possible PSG. Two other American guidelines [19, 20] recommend PSG for children who are younger than two years, have obesity or comorbidities significant to SDB, or for those without comorbidities for whom the need for surgery is uncertain.

Two European recommendations [8, 9] consider the diagnostic facilities and treatment policies across European countries. PSG is recommended for children with obstructive SDB symptoms and obesity or neuromuscular disorders, craniofacial deformities, complex abnormalities, or when the need for treatment is unclear. Although previously untreated and healthy non-obese children who are at risk for obstructive SDB due to adenotonsillar hypertrophy may be of low priority for PSG, it is recommended that alternative diagnostic examinations are carried out.

The British Thoracic Society guideline [17] found that sleep questionnaires combined with protocol-driven clinician assessment can be considered for diagnosing moderate-to-severe SDB in children of at least 2 years of age with no comorbidities. PSG is recommended if questionnaire results are inconsistent with clinical features, the child is younger than two years of age, has comorbidities, or greater diagnostic certainty is required.

The recommendations from different countries are likely influenced by the local availability of PSG, varying health insurance policies, and funding solutions. PSG is a costly and time consuming procedure, which is often not readily available. In Finland, overnight pediatric PSG is primarily available at five university hospitals, although availability can vary even among these institutions.

In clinical practice the diagnosis of pediatric SDB varies, depending on the clinic and the clinician. When a child snores and exhibits other symptoms suggestive of OSA but no obvious upper airway obstruction such as adenotonsillar hypertrophy is seen on examination, many clinicians choose to refer for PSG. However, a practical reason to refrain from requesting a PSG is the beforementioned limited capacity of sleep laboratories, which commonly results in long waiting lists. In Finland, most children are diagnosed and treated for SDB based on clinical findings and a fitting anamnesis, and no diagnostic modalities other than PSG are routinely carried out.

The objective of this study is to identify those factors that influence the decision to perform PSG in children with suspected obstructive SDB. Potential factors include cultural differences between medical specialties, comorbidities, age, sex, and reported symptoms. Given the importance of these factors in shaping the treatment pathway for children with suspected SDB and the impracticality of conducting PSG for all pediatric patients, the impact of these factors must be carefully considered. We hypothesize that the factors that influence a clinician’s decision to refer a child for PSG may be broadly generalizable, and we seek to compare our findings with existing pediatric PSG referral guidelines. Additionally, we suspect that undergoing PSG influences subsequent treatment decisions for children with obstructive SDB.

## Methods

We conducted a retrospective cohort study in the department of Otorhinolaryngology at Tampere University Hospital. Children who visited the ENT clinic of the hospital for either suspected SDB or adenotonsillar hypertrophy were included in the study.

### Patient selection

Data were extracted from the hospital’s digital patient database by three researchers between March 2023 and March 2024. The preliminary patient data search was based on surgical procedure codes (Nordic Classification of Surgical Procedures, NCSP) covering adenectomy, tonsillectomy, and tonsillotomy with or without adenectomy (EMB30, EMB10, EMB15, EMB20) and ICD-10 diagnostic codes for mouth breathing, adenotonsillar hypertrophy, and sleep apnea (ICD-10 R06.5, J35.1, J35.2, J35.3, G47.3). The children included in the study had to have one or more of the aforementioned surgical or diagnostic codes.

We only collected data on children of 16 years or younger who had attended their first appointment at the hospital’s ENT clinic between January 2014 and December 2018, resulting in 1692 patients. These patient records were reviewed manually by the three researchers. We excluded from the study all children diagnosed with central sleep apnea or those without reported or observed SDB symptoms, such as snoring, mouth breathing, apneas, and day-time tiredness, despite having the relevant ICD- or surgical codes. The final cohort consisted of 1267 children who presented with obstructive SDB symptoms during their first ENT clinic visit. Of these children, 29 were excluded from statistical analyses considering PSG being performed because these children did not follow a normal PSG taking protocol, as they were included in another ongoing study investigating childhood SDB.

### Data collection

A form for data collection was created for the REDCap software program (version 14.5.18, 2024 Vanderbilt University). Clinical data on age, sex, comorbidities of the patient, possible previous tonsillar surgeries, the origin of medical referral, reported symptoms, the clinician’s observations and findings, and the chosen treatment modalities and their outcomes were retrieved from the patient charts. For results, we gathered information on the variables of total sleep time (TST), the apnea-hypopnea index (AHI), the obstructive apnea-hypopnea index (OAHI), Oxygen 3% desaturation Index (ODI_3_), and minimum peripheral oxygen saturation (SpO_2)_. Follow-up data, including information on planned follow-up visits and revisits to the ENT clinic due to residual SDB or recurrence of the disease, were collected up to December 2023.

### Data analysis

As the significance of different comorbidities to SDB differs, we classified all children for data analysis according to their one most significant existing diagnosis. This meant that comorbidities known to increase the risk for SDB outweighed those with no or a lesser known effect on SDB. For example, juvenile rheumatoid arthritis dominated over asthma and a developmental syndrome, or an anatomical airway anomaly was considered a more significant diagnosis than other diagnoses. For further analysis, we classified the children into three groups according to the comorbidities’ level of significance to SDB: Children who were healthy or only had allergies or atopy were considered to have no significant comorbidity, whereas children with asthma, diabetes type I, neuropsychiatric and neurocognitive diagnoses, and other diagnoses were grouped into the moderate comorbidity group. Children with a developmental syndrome, anatomic anomalies of the upper airways, epilepsy, cardiovascular diseases, or juvenile rheumatoid arthritis fell into the severe comorbidity group.

The OAHI value was not determined for all children who underwent PSG at the beginning of the study period. Therefore, pediatric OSA was defined as an AHI value of ⩾ 1 episodes/h in combination with SDB symptoms, which all of our patients had. An AHI value of 1–5 episodes/h was considered a mild case, an AHI value of 5–10 episodes/h a moderate case, and an AHI value of > 10 episodes/h a severe case of OSA.

All data analyses were performed with the IBM SPSS Statistics for Windows, Version 29.0. Armonk, NY: IBM Corp. Categorical variables are reported as frequencies and percentages, and continuous variables as either means (standard deviation SD) or medians. Groups were compared using Kruskal-Wallis test, Mann-Whitney U test or Chi-Squared test. P-values of < 0.05 were considered significant. Factors (age, sex, the one most significant comorbidity, the origin of referral for SDB evaluation) that influence the decision to refer children for PSG were identified from multivariable logistic regression analysis. In the analysis, males were compared to females, children with comorbidities were compared to healthy children, and the origins of referral were compared to primary health care.

## Results

Between 2014 and 2018, 1267 children were evaluated for suspected obstructive SDB and/or adenotonsillar hypertrophy in the ENT clinic of Tampere University Hospital. Some 60% of patients were male. The mean age at the first visit was 6.5 years (median 6.0 years, SD 3.6, range 0.4–16.0). Of all the children, 94% had reported snoring, 32% mouth breathing, 32% observed apneas, and 19% day-time tiredness.

Of all the children in our study, 62% were referred for evaluation from primary health care and 23% from pediatric specialties. A further 8% of children were referred for evaluation by dentists, and 4% by other medical specialties. The referral origin was unknown in 1% of children.

A total of 65.4% of children were healthy with no comorbidities. When classified by the level of comorbidity previously described, 75.1% of children had no significant comorbidity, 15.2% had moderate comorbidity, and 9.7% had severe comorbidity.

In total, 212 children (16.7%) underwent PSG prior to any treatment. Grouped by age, sex, level of comorbidity, or the origin of medical referral, the children differed statistically significantly from each other, depending on whether or not they underwent PSG (Table 1). Children who underwent PSG were older than those who did not. Among children younger than 2 years of age, only 20% underwent PSG.

**Table 1:** Comparison of children’s characteristics based on whether they underwent PSG or not

	PSG				*p*-value
	Yes *n* = 212		No *n* = 1026		
	*n*	%	*n*	%	
Age, mean (SD), years	7.7	(4.2)	6.3	(3.4)	< 0.001
Male sex	148	70	595	58	< 0.001
Comorbidities					< 0.001
No	92	43	833	81	
Moderate	57	27	135	13	
Severe	63	30	58	6	
Origin of referral					< 0.001
Primary health care	50	24	735	72	
Dentists	1	0.5	98	10	
Pediatric specialties	158	74	128	12	
Other	2	1	52	5	
Unknown	1	0.5	13	1	

*p-value for characteristic groups differing on whether the children underwent PSG or not

The results of the univariate analyses of the children’s likelihood of undergoing PSG are presented in Table 2. The likelihood of undergoing PSG increased with age and male sex. When compared to healthy children, both children with a moderate comorbidity (OR 3.82, 95% CI 2.66–5.57, *p* <.001) and a severe comorbidity (OR 9.84, 95% CI 6.48–14.92, *p* <.001) were more likely to be referred for PSG. Certain specific comorbidities also heightened the chances of PSG being conducted.

**Table 2:** Uni- and multivariable analyses of the likelihood of the children undergoing PSG

	Univariate Analysis		Multivariable Analysis^a^	
	OR (95% CI)	*p*-value	OR (95% CI)	*p*-value
**Age**	1.11 (1.07–1.16)	< 0.001	1.14 (1.08–1.20)	< 0.001
**Male sex**	1.70 (1.24–2.34)	0.001	1.72 (1.15–2.58)	0.008
**Comorbidity**				
Healthy	1		1	
Atopy or allergies	0.68 (0.33–1.39)	0.287	0.58 (0.27–1.28)	0.179
Astma	1.65 (0.86–3.19)	0.136	0.47 (0.22–1.01)	0.054
Syndrome or an anatomical anomaly^b^	12.03 (7.41–19.54)	< 0.001	3.14 (1.71–5.75)	< 0.001
Other significant comorbidity	3.53 (2.05–6.09)	< 0.001	1.14 (0.59–2.23)	0.696
Epilepsy	15.16 (4.34–52.88)	< 0.001	3.88 (0.85–17.75)	0.080
Cardiovascular diagnosis	4.73 (1.70–13.11)	0.003	6.59 (2.05–21.13)	0.002
Juvenile rheumatoid arthritis	3.25 (0.85–12.48)	0.086	0.73 (0.15–3.62)	0.701
Neurocognitive or neuropsychiatric diagnosis	10.83 (5.40–21.71)	< 0.001	3.00 (1.21–7.46)	0.018
**Origin of referral**				
Primary health care	1		1	
Dentists	0.15 (0.02–1.10)	0.062	0.12 (0.02–0.85)	0.034
Pediatric specialties	18.15 (12.54–26.25)	< 0.001	15.00 (9.76–23.05)	< 0.001
Other	0.57 (0.13–2.39)	0.438	0.47 (0.11–2.05)	0.317
Unknown	1.13 (0.15–8.82)	0.907	1.08 (0.13–9.08)	0.943

^a^ Including age, sex, one most significant comorbidity, and origin of medical referral; ^b^ Significant developmental syndrome or an anatomical anomaly of the upper airways

The results of our multivariable analyses were mostly in line with previously presented univariate analyses: age, male sex and certain specific comorbidities (developmental syndrome or anatomical anomaly, a cardiovascular diagnosis, or a neurocognitive or neuropsychiatric diagnosis) increased the likelihood of the children undergoing PSG. When the origin of the referrals were pediatric specialties, the risk for PSG being performed increased, whereas a referral by a dentist decreased the risk. (Table 2)

Snoring did not influence the likelihood of children undergoing PSG, but apneas (OR 1.69, 95% CI 1.25–2.29, *p* <.001) and day-time tiredness (OR 3.97, 95% CI 2.87–5.49, *p* <.001) increased the likelihood of PSG. However, children with mouth breathing were less likely to undergo PSG (OR 0.58, 95% CI 0.41–0.82, *p* =.002) than those with no mouth breathing.

Children differed from each other in origins of referral. When compared by comorbidity levels, those children referred by pediatric specialties had the highest percentage of moderate (33%) or severe (28%) comorbidities, whereas 86% of the children referred from primary health care and 89% of children referred from dentistry were healthy (*p* <.001). Children referred from dentistry had the most mouth breathing (55%) and the least observed apneas (9%) and day-time tiredness (9%), whereas children referred from pediatric specialties had the most apneas (40%) and day-time tiredness (32%). Children referred by dentistry were also significantly older (mean 9.2 years, SD 2.3, *p* <.001) than children referred from primary health care (mean 6.1 years, SD 3.3) or children from pediatric specialties (mean 6.8 years, SD 4.1).

The PSG results of the children are presented in Table 3. Of those children who underwent PSG, 13% had normal PSG results, whereas 37% had a mild, 23% a moderate, and 26% a severe case of OSA. The PSG results had no correlations with age and no differences according to sex were found. After Bonferroni’s correction, the OAHI values were found to differ statistically significantly when children referred by pediatric specialties were compared to those from primary health care (p-value 0.018). Indeed, children referred by pediatric specialties had a higher OAHI value (mean 10.1, median 4.6, SD 17.1, range, 0.0-108.0) than those from primary health care (OAHI mean 7.9, median 1.9, SD 21.2, range, 0.0-115.2).

**Table 3:** PSG results for all children based on level of comorbidity

		All Children	No significant comorbidity	Moderate comorbidity	Severe Comorbidity	*P*-value
		*n* = 210	*n* = 92	*n* = 56	*n* = 62	
TST	Mean (SD), min Median, min	509.6 (66.9) 517.3	519.2 (61.3) 523.3	495.1 (69.4) 503.8	508.5 (71.1) 516.5	0.115
AHI	Mean (SD) Median	10.5 (17.8) 4.9	7.8 (14.4) 3.9	16.3 (24.8) 5.6	9.4 (12.0) 6.0	0.024
OAHI	Mean (SD) Median	9.5 (18.0) 3.7	6.7 (16.0) 2.4	16.2 (25.1) 5.4	8.5 (12.3) 5.2	0.003
ODI_3_	Mean (SD), % Median, %	6.2 (12.9) 1.9	4.7 (12.2) 1.7	9.7 (17.0) 2.1	5.5 (8.5) 2.2	0.147
SpO_2_	Mean (SD), % Median, %	89.7 (5.9) 91.0	90.5 (4.5) 91.0	89.4 (5.9) 90.6	88.8 (7.6) 91.0	0.489

The comorbidities of the children affected the PSG results (Table 3). The AHI and OAHI values were more severe in those children with moderate or severe comorbidities than in children with no significant comorbidities. There were no statistically significant differences between the moderate and severe comorbidity groups in any of the PSG result parameters. However, pairwise comparisons showed statistically significant differences between children without comorbidities and those with comorbidities in the AHI value (*p* =.017 for moderate and *p* =.029 for severe comorbidity) and in the OAHI value (*p* =.002 for moderate and *p* =.015 for severe comorbidity).

Finally, we found that children who underwent PSG differed from those diagnosed clinically when it came to the chosen treatment modalities (Table 4). Children who underwent PSG were less likely to need any further treatment and more likely to be treated conservatively than those who did not undergo PSG.

**Table 4:** Chosen treatment modalities and whether the children underwent first line PSG or not

Chosen treatment**	All children	PSG	
	*N* = 1238	YES *n* = 172	NO *n* = 1026
	%	%	%
Watchful waiting	8.6	7.6	9.1
Operative treatment^a^	81.6	77.9	85.4
Other^b^	3.2	7.6	2.6
Treatment not needed	3.4	7.0	2.9

* (*p* <.001) When children were compared based on whether they underwent PSG or not and treatment modalities

** Children for whom the information on the chosen treatment could not be found are excluded from the analysis

^a^Adenectomy, tonsillectomy, or tonsillotomy with or without adenectomy

^b^Intranasal steroid, oral or topical antihistamine, other

## Discussion

In our study, age, sex, comorbidities, symptoms of OSA, and the origin of the medical referral all influenced the likelihood of the children undergoing PSG. This highlights the importance of a single clinician’s decision making, as we think it is likely that this subjectivity is broadly generalizable and not limited to the clinicians of a specific hospital.

### Factors that influence the decision to perform PSG

Males had a higher likelihood of undergoing PSG than females in our study. Some studies suggest that male children are more likely to have OSA [21, 22], although this finding is not universally supported across all studies [23]. In existing recommendations, sex is not viewed as a risk factor for pediatric OSA that would encourage performing PSG. As sex did not influence the PSG results in our study, it raises the question as to whether PSG is genuinely more necessary for males, and whether female children are being underdiagnosed. As obstructive sleep apnea in adults is more common in men than women [21], this finding might lead to the perception that pediatric OSA is also more of a male issue, thereby influencing diagnostic decisions.

Some of the existing guidelines recommend referring children younger than 2 years for PSG [17, 19, 20]. In our study, however, only a minority (20%) of childen younger than 2 years underwent PSG, and the risk for children to undergo PSG rose slightly as they aged. It is, of course, possible that age alone is not considered a factor significant enough to justify performing PSG in clinical decision making. However, as these results conflict with previous recommendations, it further underlines the urgent need for a universal guideline on this matter.

Developmental syndromes and anatomical anomalies, cardiovascular diagnoses, and neuropsychiatric and neurocognitive diagnoses increased the likelihood of PSG being performed. This is mainly consistent with existing recommendations, as children with certain significant comorbidities are more at risk for having OSA. This, in turn, aligns with our study’s findings, where the PSG results were worse in proportion to the severity of the children’s comorbidities. Some comorbidities that are known to be risk factors for OSA (juvenile rheumatoid arthritis and epilepsy [9, 22]) were left statistically insignificant considering the likelihood of PSG, which was most likely due to very small group sizes.

It is noteworthy that children with a neurocognitive or neuropsychiatric diagnosis, such as ADHD, an autism spectrum disorder, or developmental delay, were more likely to undergo PSG. Because these conditions can co-occur with obstructive SDB, and their symptoms partly overlap with SDB’s, it is possible that SDB in children is sometimes misdiagnosed as ADHD [23]. This would encourage clinicians to screen these children for SDB with PSG. Currently, existing recommendations do not specifically address neuropsychiatric or neurocognitive diagnoses in relation to the use of PSG. However, this finding raises the question as to whether guidelines should address this issue, especially as the number of children being diagnosed and treated for ADHD is rising [24–26].

In our study, symptoms of more severe OSA (apneas and day-time tiredness) increased the likelihood of PSG being performed. This finding is consistent with current guidelines, as they recommend PSG for those suspected of having severe OSA. However, another obstructive SDB symptom, mouth breathing, reduced the likelihood of PSG, and snoring did not influence the likelihood of PSG at all, highlighting the subjective nature of clinically assessing the severity of potential SDB. It is possible that because mouth breathing is viewed as an obvious sign of OSA, PSG is deemed unnecessary. Alternatively, mouth breathing could also be considered to have such a significant negative impact on dental health and occlusion development and possibly halitosis that as a symptom alone it is enough to promote treatment decisions.

It is of note that for the majority of children in our study, the size of the adenotonsillar tissue had been assessed only descriptively, without standardized or systematic grading, making it challenging to evaluate the impact of adenotonsillar size assessment on the decision to refer for PSG. However, when adenotonsillar tissue appears small in the presence of OSA symptoms, clinicians may be more likely to refer for PSG. Further prospective studies are needed on this topic, taking into account the potential influence of adenotonsillar tissue size on clinical decision-making.

Adjusted for age, sex, and comorbidities, children referred from pediatric specialties were more likely to undergo PSG, while those referred by dentists were less likely to do so. Children referred from pediatric specialties also had slightly higher OAHI values, suggesting that these children were more likely to have more severe OSA. This aligns with the higher prevalence of significant comorbidities in this group, supporting the decision to perform PSG. Children from pediatric specialties also had more day-time tiredness and reported apneas, which are both symptoms associated with an increased likelihood of PSG being performed. Children referred by dentists were, in turn, mainly healthy and had the highest rates of mouth breathing. They were also older than other children, which is logical as children are usually referred by a dentist for ENT evaluation when orthodontic treatment is considered, typically later in childhood. It is possible, therefore, that children referred by dentists are evaluated by the clinician with a focus more on dental occlusion and mouth breathing symptoms than screening for SDB. Overall, the likelihood of children undergoing PSG is influenced, in part, by the origin of their medical referral, suggesting that clinical cultures and differences between specialities contribute to this variation, which again highlights the need for standardized guidelines for PSG use.

### The effect of undergoing PSG on treatment choices

A significant finding in our study was that undergoing PSG subsequently affected the children’s treatment choices. Children who underwent PSG were more likely to either need no treatment at all (7% vs. 2.9%) or to be treated conservatively. In contrast, those children who did not undergo PSG were more often operated on (85.4% vs. 77.9%). As undergoing PSG resulted in a higher rate of less burdensome treatments, it raises the question as to whether we are overtreating children with suspected SDB, as PSG reliably reduces wrong positive diagnoses, and thereby reduces unnecessary treatments.

### Strengths and limitations

This is a retrospective study based on patient records. Thus, the data on patients were limited to what the treating physician had recorded, and not all data are retrievable for all patients with a study design of this nature. Additionally, we were not able to consider childhood obesity in our study, as information on weight, ISO-BMI%, and height was not obtainable for all the patients in our study. As obesity is known to be a significant risk factor for childhood obstructive SDB, this can be considered a limitation of the study. Also, we only used the patient records of Tampere University Hospital, so if the patients in this study were subsequently treated at other hospitals, information on this later treatment was not included in our data.

A main strength of this study is the reasonable size of our cohort of children with suspected obstructive SDB and/or adenotonsillar hypertrophy. We also have a respectable cohort of children who underwent PSG as a first line procedure.

## Conclusion

The clinician’s decision to refer a child for PSG is susceptible to subjectivity, and the factors influencing this decision are likely broadly generalizable. Furthermore, while a previous guideline recommends PSG for all regularly snoring children, children with mouth breathing are not thought to need further examination. Thus, a universal guideline for referring children with suspected SDB for PSG is needed to better support clinical decision making. This would not only standardize the SDB diagnostic pathway, but also potentially reduce the need for surgeries or further follow-up, as having PSG performed reduces the need for either.

## Electronic supplementary material

Below is the link to the electronic supplementary material.

Supplementary Material 1
