# Supplementary material for: Suspected pediatric sleep disordered breathing – when do we perform polysomnography?
Source: Eur Arch Otorhinolaryngol. 2025 May 29;283(2):1271–8. doi: 10.1007/s00405-025-09480-z (PMC12987901; doi:10.1007/s00405-025-09480-z)
Supplement: Supplementary file 1 — Supplementary Material 1 [file 405_2025_9480_MOESM1_ESM.docx]

March 31st 2025

Ella Ruikka

MD

Tampere University Hospital

P.O. Box 2000,

33520 Tampere,

Finland

Dear Editors,

We wish to submit our original research article, ”Suspected pediatric sleep disordered breathing – when do we perform polysomnography?” for consideration for publication in European Archives of Oto-Rhino-Laryngology. We affirm that this work is original, has not been published previously and is not currently under consideration for publication elsewhere.

In this retrospective cohort study on children suspected of having sleep disordered breathing, we identified factors that influence clinicians’ decisions to refer children for polysomnography. Our findings indicate that multiple factors—including age, sex, comorbidities, certain symptoms, and the children’s origin of medical referral—influence the likelihood of undergoing polysomnography. Notably, we also found that children who underwent polysomnography were more likely to be treated conservatively than those diagnosed clinically.

Our study’s findings show that the clinician’s decision to refer a child for polysomnography is susceptible to subjectivity. Given that polysomnography is a costly and time-consuming procedure that is not always readily available, understanding the factors influencing referral decisions is essential. Furthermore, our study suggests that undergoing polysomnography subsequently affected the children’s treatment choices, potentially reducing the need for surgical interventions or prolonged follow-up.

Our findings align with the few existing recommendations for polysomnography referrals in some respects; however, certain factors we identified are not explicitly addressed in current guidelines. Our study’s findings highlight the need for a universal guideline for referring children with suspected sleep disordered breathing for polysomnography to better support clinical decision making, and to standardize the diagnostic and treatment pathways for these children.

The corresponding author, Ella Ruikka has received funding for this research from the Finnish ORL-HNS Foundation (grant number 20250024). We have no other conflicts of interest to declare.

Please address all correspondence concerning this manuscript to me at [ella.ruikka@tuni.fi](mailto:ella.ruikka@tuni.fi).

Thank you for your time and consideration of this manuscript.

Sincerely,
Ella Ruikka

[ella.ruikka@tuni.fi](mailto:ella.ruikka@tuni.fi)
